# Lower lid entropion correction with botulinum toxin injection

**DOI:** 10.4103/0974-620X.71909

**Published:** 2010

**Authors:** Amarendra Deka, S. P. Saikia

**Affiliations:** Department of Oculoplasty, Bawri Nethralaya, Shillong, India

Sir,

Entropion is the inward turning of the eyelid and is most commonly seen as an aging phenomenon produced by attenuation or detachment of the lower eyelid retractor and associated with horizontal eyelid laxity. Although surgical treatment is more reliable and remains the treatment of choice, various medical treatments such as tissue glue and botulinum toxin have been advocated.

We report the results of treatment of lower lid entropion with botulinum toxin A.

Seventeen patients with senile entropion, one patient with congenital entropion and two patients with congenital entropion with corneal epithelial defect were included for this study. Patients were explained about the effect and side effects of the toxin. Informed consent was taken.

All the cases had entropion more than 6 months, and two patients had previous entropion surgery. Thirteen patients that included two children had bilateral entropion.

**Figure 1:**
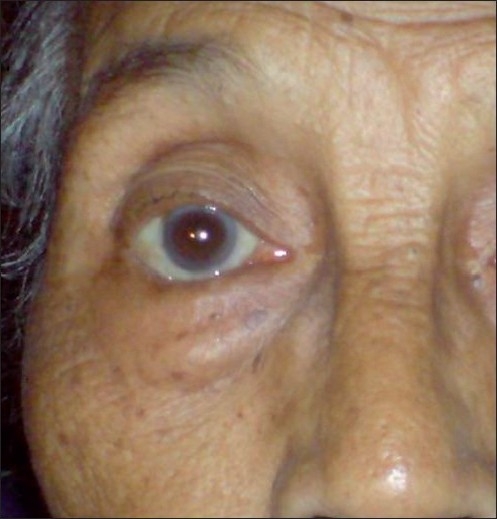
Preinjection

**Figure 2:**
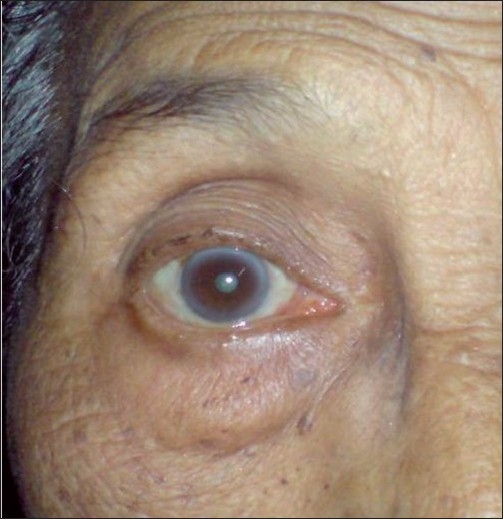
Postinjection

The toxin (Botox®, Allergan Corporation, Irvine, CA, USA) that was supplied in a vial contained 100 units of freeze-dried botulinum toxin A. This was reconstituted and diluted with 2 mL of saline to make it five units in 0.1 ml. The reconstituted toxin was injected subcutaneously[1] over the orbicularis oculi muscle about 3–4 mm below the eyelash margin of lower lid at three sites with a 30-gauge needle attached to a 1-mL syringe. Most of the patients were re-examined daily for 1 week, then biweekly for 6 months to know the duration of relief. The ethics review board of Bawri Nethralaya approved this study [Figures 1 and 2].

In 19 patients including one case that had previous surgery, improvement was immediate and sustained. However, one other patient who had previous entropion surgery did not improve as expected. The volume on toxin that tended to pull the eyelash margin away from the globe resulted in immediate improvement. The actual effect on the eyelid margin was visible within 3 to 4 days of infection. The duration of improvement varied from 8 to 16 weeks. However, a 4-year-old child showed sustained improvement for a period of 26 weeks [Table 1]. Corneal defects in two children healed following correction of entropion. No side effect was noticed.

**Table 1:** Patients profile

*Age group*	*Diagnosis*	*Sex*		*Doses (units)*	*Previous operation*	*Duration of relief after first dose (weeks)*
		*M*	*F*			
0–9	Congenital entropion with corneal epithelial defect		1	7.5	–	26
10–19	Congenital entropion and congenital entropion with corneal ulcer	2		7.5	–	8–12
40–49	Senile entropion	0	1	15	1	12–15
50–59	Senile entropion	3	3	15		13–16
60–69	Senile entropion	4	3	15	1	14–16
70–79	Senile entropion	1	2	15	1	13–15

Botulinum toxin A is an exotoxin produced by Clostridium botulinum, a gram-positive anerobic bacterium. The toxin inhibits the release of acetylcholine at neuromuscular junctions affecting temporary paralysis of targeted muscle. Recovery of muscle action occurs because of axonal sprouting and formation of new neuromuscular junction.[2,3]

Although surgical correction of senile entropion is definitive and permanent, botulinum toxin injection results in temporary correction of senile entropion as documented in a previous study.[1,4] It is a safe and quick outpatient procedure, results in temporary but immediate improvement of the condition. The toxin has a longer effect in patients with less lower lid laxity.[1]

In summary, although surgical treatment should be the choice of treatment for entropion, botulinum toxin is a safe and effective procedure for correction of some cases of senile and congenital entropion. It is very useful in children having congenital entropion with corneal defect, where immediate improvement is essential.[5]
